# Progress toward Room-Temperature Synthesis and Functionalization of Iron-Oxide Nanoparticles

**DOI:** 10.3390/ijms23158279

**Published:** 2022-07-27

**Authors:** Diego A. Flores-Cano, Noemi-Raquel Checca-Huaman, Isabel-Liz Castro-Merino, Camila N. Pinotti, Edson C. Passamani, Fred Jochen Litterst, Juan A. Ramos-Guivar

**Affiliations:** 1 Grupo de Investigación de Nanotecnología Aplicada para Biorremediación Ambiental, Energía, Biomedicina y Agricultura (NANOTECH), Facultad de Ciencias Físicas, Universidad Nacional Mayor de San Marcos, Av. Venezuela Cdra 34 S/N, Ciudad Universitaria, Lima 15081, Peru; diego.flores4@unmsm.edu.pe; 2 Centro Brasileiro de Pesquisas Físicas (CBPF), R. Xavier Sigaud, 150, Urca, Rio de Janeiro 22290-180, Brazil; noemiraquelchecca@gmail.com (N.-R.C.-H.); isabel5cas@hotmail.com (I.-L.C.-M.); 3 Physics Department, Federal University of Espírito Santo, Vitória 29075-910, Brazil; camilapinotti23@gmail.com (C.N.P.); passamaniec@yahoo.com.br (E.C.P.); 4 Institut für Physik der Kondensierten Materie, Technische Universität Braunschweig, 38106 Braunschweig, Germany; j.litterst@tu-braunschweig.de

**Keywords:** iron oxide, nanoparticles, room-temperature synthesis, functionalization

## Abstract

Novel magnetic nanohybrids composed of nanomaghemite covered by organic molecules were successfully synthesized at room temperature with different functionalization agents (sodium polystyrene sulfonate, oxalic acid, and cetyltrimethylammonium bromide) in low and high concentrations. Structural, vibrational, morphological, electron energy-loss spectroscopy, magnetic, and Mössbauer characterizations unraveled the presence of mainly cubic inverse spinel maghemite (γ-Fe_2_O_3_), whilst X-ray diffraction and ^57^Fe Mössbauer spectroscopy showed that most samples contain a minor amount of goethite phase (α-FeOOH). Raman analysis at different laser power revealed a threshold value of 0.83 mW for all samples, for which the γ-Fe_2_O_3_ to α-Fe_2_O_3_ phase transition was observed. Imaging microscopy revealed controlled-size morphologies of nanoparticles, with sizes in the range from 8 to 12 nm. Organic functionalization of the magnetic nanoparticles was demonstrated by vibrational and thermogravimetric measurements. For some samples, Raman, magnetic, and Mössbauer measurements suggested an even more complex core-shell-like configuration, with a thin shell containing magnetite (Fe_3_O_4_) covering the γ-Fe_2_O_3_ surface, thus causing an increase in the saturation magnetization of approximately 11% against nanomaghemite. Field cooling hysteresis curves at 5 K did not evidence an exchange bias effect, confirming that the goethite phase is not directly interacting magnetically with the functionalized maghemite nanoparticles. These magnetic nanohybrids may be suitable for applications in effluent remediation and biomedicine.

## 1. Introduction

The relevance of nanomaterials in environmental fields such as nano-agriculture and nano-remediation has been increasing over the last decades [1]. Particularly, magnetic nanomaterials based on iron-oxides (magnetite-Fe_3_O_4_ and maghemite-γ-Fe_2_O_3_) outstand due to their large specific surface area, tunable structures, eco-friendly characteristics, high catalytic response, and magnetic properties, which make them attractive materials in the above and many more fields [2,3,4]. For instance, among the benefits of Fe_3_O_4_ nanoparticles (NPs), it highlights their absorbent properties, acting as blocking agents, in the Cu transport mobility in polluted soils or crops [5]. Moreover, Moringa oleifera treatment with Fe_3_O_4_ NPs improved its growth by reducing the salinity effects [6]. On the other hand, experiments with soybeans demonstrated the outstanding potential of γ-Fe_2_O_3_ NPs as sustainable and highly efficient crop fertilizers, especially NPs with the smallest sizes [7]. In water remediation, several bare and functionalized γ-Fe_2_O_3_ and Fe_3_O_4_ NPs exhibit remarkable removal efficiencies of toxic metal ionic species and different dye types [2,8].

There are several works reported in the literature that deal with the synthesis parameters and their optimizations to produce iron-oxide nanoparticles for different applications [2,3,4,5,6,7,8,9,10]. In particular, a current review [2] summarizes the principal issues of these nanomaterials, where features such as scalability, production, and application costs are discussed because they are some of the main challenges for their industrial-level implementation. Consequently, synthesis and functionalization costs of magnetic nanoparticles (MNPs) vary depending on the production route. Among several physical and chemical routes applied to synthetize MNPs, we can mention the co-precipitation method [2], the arc discharge plasma method [9], sonication, and pyrolysis [10], but the co-precipitation method excels among other routes due to its high resultant product mass in short periods [2]. However, even though this method often allows functionalization in simple steps, it still requires energy for thermal treatment and surface activation processes. This means that increasing the temperature to 80 °C, for example, will result in better dispersed samples with homogeneous morphologies. Other important parameters in the co-precipitation method are the pH, medium ionic strength, and molar ratio [2]. This last parameter can significantly be affected upon synthesis in the presence of inorganic/organic frameworks or supporting matrices. To overcome this and to contribute to their scalability, the room temperature (RT) functionalization needs to be explored in the presence of several matrices in order to produce magnetic nanohybrids composed of iron-oxides recovered by a specific organic substance that can be applied for a certain purpose.

Thus, in this work, iron-oxide MNPs were successfully functionalized at RT using the co-precipitation method. Specifically, six nanohybrid samples were synthesized using sodium polystyrene sulfonate (PSS), oxalic acid (OA), and cetyltrimethylammonium ammonium bromide (CTAB) organic substances at two different concentrations with the aim to explore the stoichiometry, crystallinity, thermal stability, particle size control, and magnetic properties.

## 2. Results and Discussion

### 2.1. X-ray Diffraction Analysis and Rietveld Refinement

**Figure 1:**
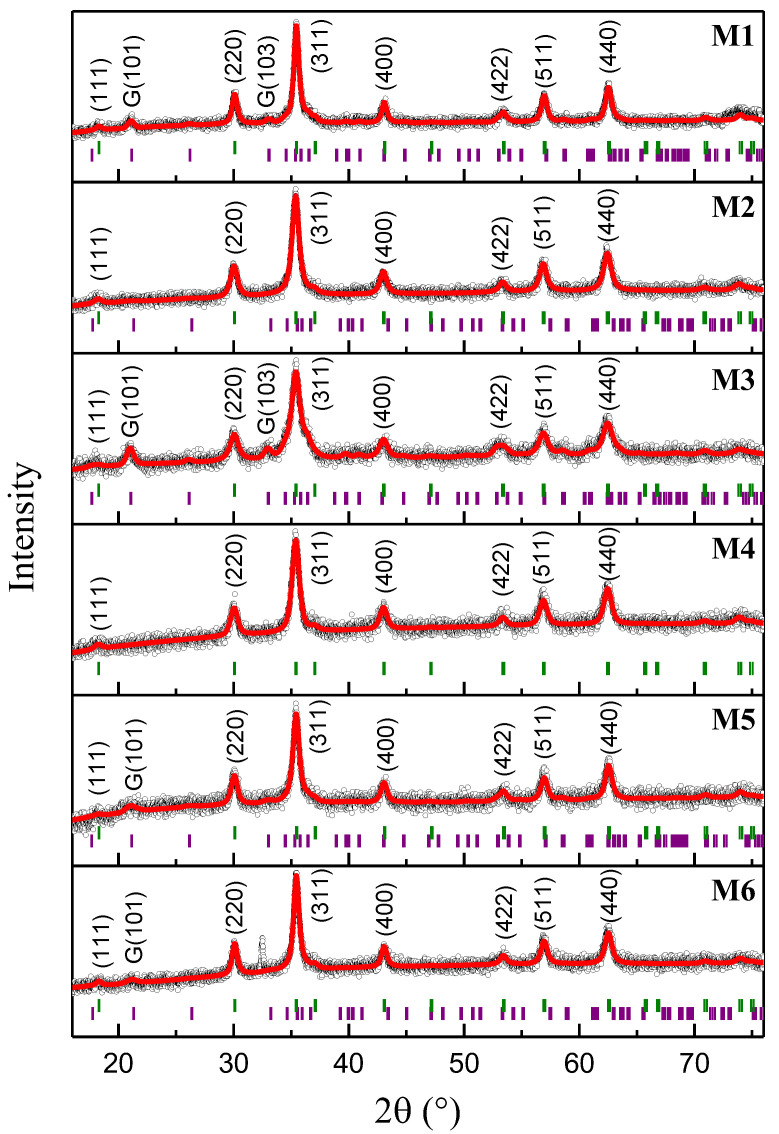
Rietveld refined XRD patterns of the M1–M6 samples. Black circles are the experimental data, red lines indicate calculated XRD pattern, and olive and purple vertical lines represent the Bragg positions of the γ-Fe_2_O_3_ and α-FeOOH phases, respectively.

Figure 1 shows the Rietveld refined diffractograms of each sample. They present Miller planes corresponding to a γ-Fe_2_O_3_ main phase [11] and an α-FeOOH secondary phase [12,13], except for the M4 sample, which only shows the γ-Fe_2_O_3_ phase (no organic phase was detected by XRD experiments). The modeled space group of the γ-Fe_2_O_3_ phase is Fd$\overset{–}{3}$m, as confirmed by the presence of the (111) plane around 18°. It suggests a random distribution of vacancies in the γ-Fe_2_O_3_ unit cell [14,15], consequently its crystalline structure corresponds to an inverse spinel cubic structure with Fe atoms distributed in A (tetrahedral) and B (octahedral) sites [2]. The space group of α-FeOOH is Pbnm, an orthorhombic structure with Fe atoms located in octahedral sites [13]. However, it should be mentioned that, as shown by the Bragg positions in Figure 1, the peaks of both phases overlap several times (most of the minor contributions of α-FeOOH are screened by γ-Fe_2_O_3_ planes and the background noise). The most notable Miller planes of α-FeOOH, (111) and (103), are located at around 21° and 33°, respectively. Table S1 shows the refinement and statistical parameters of each sample. From this table, it can be observed that the lattice parameters of the γ-Fe_2_O_3_ phase remain ca. (8.40 + 0.01) Å for all samples. Table S2 presents the microstructural parameters and contributions of each phase. The phase contribution values express well the α-FeOOH peaks on each diffractogram. The crystallite size of both phases corresponds to nanometric Fe-oxides. This information can be enlightening when correlated with the magnetic behavior of the γ-Fe_2_O_3_ phase, since it highly depends on its particle size [2]. It must be observed that the M3 sample exhibits a remarkably smaller crystallite size and a higher percentage of α-FeOOH contribution, presumably because of pouring less NH_4_OH during the co-precipitation synthesis, consequently a more disordered contribution is expected for the α-FeOOH phase.

### 2.2. µ-Raman Analysis

**Figure 2:**
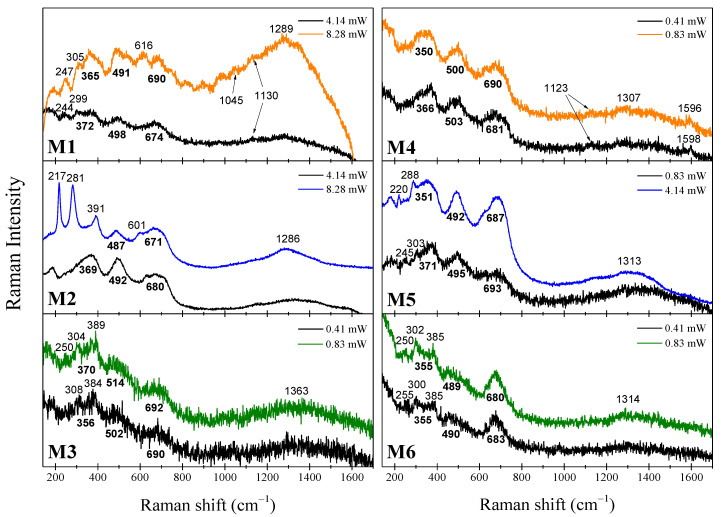
Raman spectra of the samples M1-M6 at different laser power values. The Raman shift of each mode is indicated in cm^−1^ and the γ-Fe_2_O_3_ broad modes are bolded and put below the corresponding peaks. The equal colors of spectra set (orange, blue, and olive) represent the related functionalizing agents of the samples, i.e., PSS, OA, and CTAB.

In Figure 2, the Raman spectra of each sample taken at two different laser powers are shown. It should first be mentioned that the influence of laser power is manifested on the spectra comparing the data recorded individually after the progressive increase in laser power. Considering the XRD results that mainly show the predominance of the γ-Fe_2_O_3_ phase, their vibrational modes are identified by the broad peaks at approximately 361, 496, and 687 cm^−1^ for all the samples [14,16,17]. At this point, we must highlight that pure nanomaghemite exhibits an optical Raman mode at 720 cm^−1^ [14], and in our samples the main peak is located at ~690 cm^−1^. Thus, this result is strong evidence for a core-shell configuration since nanomagnetite has a Raman mode at 670 cm^−1^ [16], but an agreement among the studied techniques must be reached. Hence, we will return to this later when discussing the Mössbauer and magnetization results. In addition, it should be mentioned that the peaks of the α-FeOOH phase are located at around 250, 302, and 385 cm^−1^, as can be seen in spectra of the M1, M3, M5, and M6 samples [18,19]. The α-Fe_2_O_3_ modes started to show up at higher laser power values for the M1, M2, and M5 samples due to a structural phase transformation of the γ-Fe_2_O_3_ and α-FeOOH phases into the α-Fe_2_O_3_ one induced by thermal effects of the laser [14,16,17]. The broad band at approximately 1300 cm^−1^ observed in all the spectra is often found in Fe-oxides [20]. The functionalization with PSS of the M1 and M4 samples are corroborated by the shifted PSS bands at 1045, 1123–1130, and 1596–1598 cm^−1^ [21]. Neither the Raman band of the OA nor CTAB was found in the Raman spectra [22,23]. The positions of the found vibrational modes of each sample are summarized and ordered by phases and laser power values in Table S3.

These results shed some light on the thermal stability of the samples, specifically the γ-Fe_2_O_3_ NPs related to their functionalization. Up to 0.83 mW, all samples appear to be thermally stable because no evidence of the α-Fe_2_O_3_ modes is detected within our experimental resolution. Thus, this laser power would be an adequate threshold for Raman studies on these composites searching for the non-degradation of the γ-Fe_2_O_3_, i.e., for avoiding the γ to α phase transition. In another study, under similar instrumental conditions, the threshold laser power value was determined to be 0.10 mW for pure γ-Fe_2_O_3_ [14]. Thus, our results would suggest that the organic functionalizing agents used in this research, contribute positively to the thermal stability (×8) of the γ-Fe_2_O_3_ NPs, even if we have a small contribution of the α-FeOOH phase. As laser power reaches 8.28 mW on the M1 sample, the γ-Fe_2_O_3_ modes at 372, 498, and 674 cm^−1^ start to fall apart and shift to lower wave numbers. An α-Fe_2_O_3_ peak at 616 cm^−1^ arises, suggesting the thermal transformation of the γ-Fe_2_O_3_ to α-Fe_2_O_3_. At the same time, the α-FeOOH modes remained, even at 8.28 mW. This phase transformation can also be detected in the OA functionalized samples. However, as observed in Raman spectra of the M5 sample, the α-FeOOH phase transforms first into α-Fe_2_O_3_ due to local thermal treatment [24]. This is evidenced by the disappearance of the α-FeOOH modes at 245 and 303 cm^−1^ in exchange for the arising of the α-Fe_2_O_3_ modes at 220 and 288 cm^−1^. This behavior seems to be caused by OA functionalization since PSS functionalized samples, as M1, have maintained their α-FeOOH contributions, while the γ-Fe_2_O_3_ phase has transformed into α-Fe_2_O_3_. In other words, the α-FeOOH phase has more thermal stability when functionalized with the PSS than with the OA. CTAB functionalized samples did not exhibit structural phase transformations up to 0.83 mW. Nevertheless, all the samples were fully transformed into α-Fe_2_O_3_ after being burned at 82.8 mW, as shown in Figure S1, where the seven characteristic α-Fe_2_O_3_ bands were noticed, and no γ-Fe_2_O_3_ phase is remained.

### 2.3. FTIR Analysis

**Figure 3:**
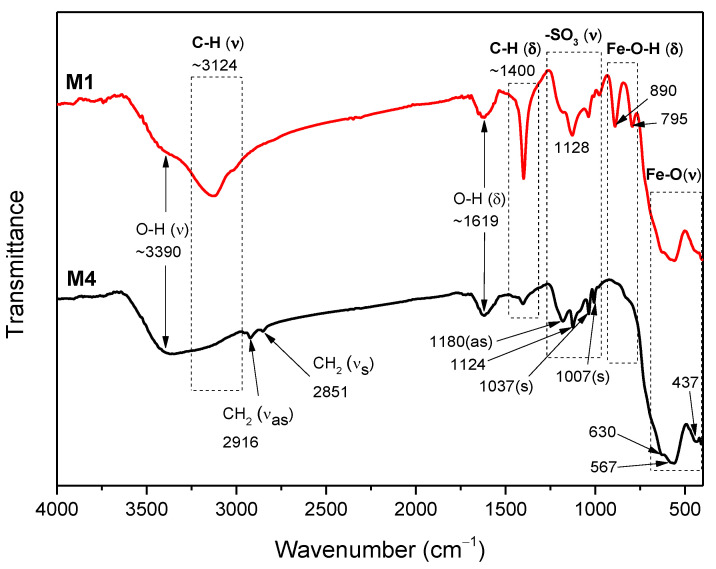
FTIR spectra of the M1 and M4 samples. The peaks positions are indicated in cm^−1^.

Figure 3 shows the IR spectra of the M1 and M4 samples. Fe-O stretching vibrations correlated with nano γ-Fe_2_O_3_ are located at 630, 567, and 437 cm^−1^ [25,26]. The peaks at 890 and 795 cm^−1^ correspond to the Fe-O-H bending vibration bands, a characteristic of the α-FeOOH phase [27,28,29]. Additionally, in accordance with the DRX and Raman results, the M4 sample does not present the α-FeOOH phase. The functionalization in both cases is verified by the presence of -SO_3_ characteristic peaks of PSS between 1000 and 1250 cm^−1^ [30,31,32]. On the other hand, the influence of the PSS concentration during the synthesis process, can be observed in the M4 sample by the CH_2_ symmetric and asymmetric stretching vibrations at 2851 and 2916 cm^−1^, respectively [31].

**Figure 4:**
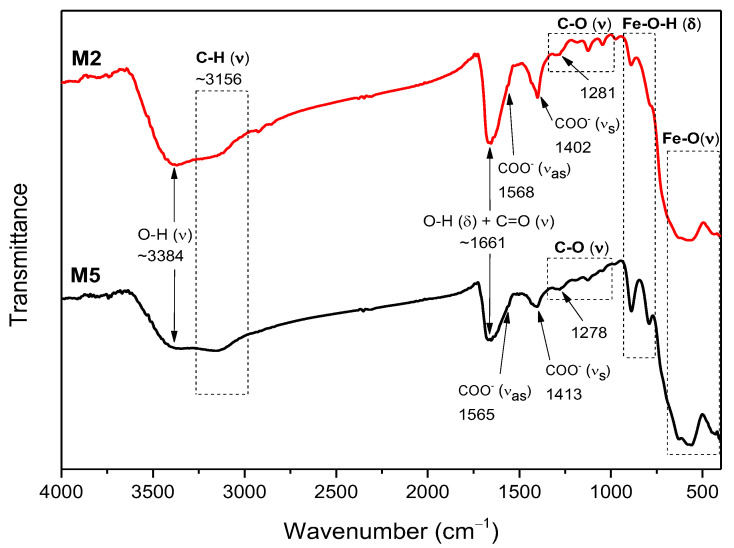
FTIR spectra of the M2 and M5 samples. The peaks positions are indicated in cm^−1^.

The M2 and M5 IR spectra are presented in Figure 4. Once again, the Fe-O stretching and Fe-O-H bending bands due to the γ-Fe_2_O_3_ and α-FeOOH are located at the 410–900 cm^−1^ range. The OA functionalization is verified by the presence of carboxyl groups as a redshifted C=O stretching band at ~1661 cm^−1^ (overlapped with the O-H bending vibration band from physiosorbed water molecules on nano γ-Fe_2_O_3_ NPs), and the COO^−^ symmetric and asymmetric stretching bands at 1413 and 1565 cm^−1^ for theM5 sample, and 1402 and 1568 cm^−1^ for the M2 sample, respectively [33,34,35,36,37]. The redshift of the C=O band corresponds to the chemisorption of the carboxyl groups onto the ionic Fe surface [33]. It can be noticed that the COO^−^ group symmetric stretching bands are overlayed with that of the C-H bending vibration band [35].

Figure S2 shows the IR spectra of the M3 and M6 samples. The Fe−O and Fe−O−H vibration bands are located at the same range as observed for all samples, and the CTAB coating is verified by the presence of CH_2_ asymmetrical and symmetrical stretching vibration bands at 2948 and 2850 cm^−1^, respectively [38,39]. The short vibration bands at 1467 cm^−1^ in the M3 sample and 1461 cm^−1^ in the M6 sample correspond to the N^+^−CH_3_ symmetrical vibration [39]. They suggest the prevalence of CTAB molecules, which have not interacted with the nano γ-Fe_2_O_3_ surface [40,41]. At ~1625 cm^−1^, the O−H bending vibration band appears to overlap with the N^+^-CH_3_ asymmetrical vibration of the CTAB [39].

In all samples, the O-H stretching and bending vibration bands due to the physiosorbed water molecules onto nano γ-Fe_2_O_3_ are observed as a broad peak centered around 3300–3500 cm^−1^ and a medium one between 1619 and 1661 cm^−1^, respectively [14,34]. Additionally, the broad peaks centered near 3124–3156 cm^−1^ due to the C-H stretching vibration [37] and the bands between 1400 and 1500 cm^−1^ due to the C-H bending vibration [40] are also an indicative for the prevalence of organic molecules, such as PSS, OA, and CTAB. In the 1000–1300 cm^−1^ range, carboxyl C-O groups are identified along with other small peaks. They could suggest the formation of alkoxy, epoxy, and carboxyl groups, sometimes overlapped with other bands [14,39,42]. Finally, it must be mentioned that peaks at approximately 1400 cm^−1^ could contain contributions of N bonded ion groups such as NH_3_ and NH_4_ [37,43], which could arise from the NH_4_OH used in the synthesis process of all the samples. The estimated positions of each vibration band are summarized in Table S4.

### 2.4. TG Analysis

**Figure 5:**
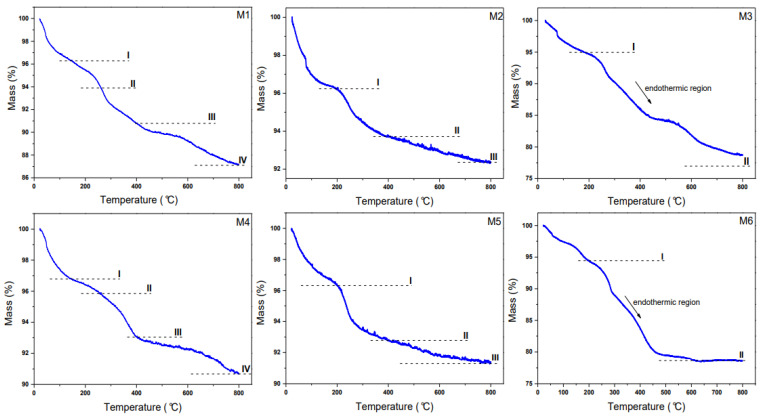
TG curves obtained from RT up to 800 °C for the M1-M6 samples. The steps described in the text are defined in each TG curve.

Thermogravimetric measurements are displayed in Figure 5. For the M1 sample (1 µM PSS), a four-steps curve is observed, where the first and second steps, located at 145 °C and 261 °C, are assigned to physiosorbed and crystallized water, respectively [44]. The third mass lost at ~ 400 °C is assigned to two distinct steps: (i) the goethite transformation into hematite (this endothermic transformation occurs at a temperature of 324–350 °C [45]) and (ii) the decomposition of PSS in the interval of 200–250 °C. This PSS mass loss (chain carbon decomposition) continues till 800 °C, with a total percentage value of 12.9%. The same decay behavior was noticed for the M4 sample, (2 µM PSS). However, the total weight loss in this last case was 9.3%. It seems that for higher concentrations, the loaded PSS is desorbed onto the γ-Fe_2_O_3_ surface, reaching a saturated surface state. These results agree with the work of Chen et al. [46], who have reported a total mass loss of 17% for PSS (30% *v*/*v*) at 65 °C.

With the aim of studying the TG curves of OA coating of the NPs, it is worth mentioning that the fatty acid decomposition has previously been explored and that three steps have been found [47]. The literature suggests that they are: (i) carboxylic acid degradation (200–300 °C), (ii) carboxylic acid desorption (400–600 °C), and (iii) residual carbon formation (600 to 800 °C). Therefore, we secondly characterized the 14 and 28 mM for the M2 and M5 (OA) samples, where a total mass loss of 7.9% and 9% was respectively found. Notably, both TG curves exhibited three steps with values of 4%, 2.4–3.5%, and 1.5%, where the first two losses are found in the region of 20–400 °C. Here, the first one is related to physisorption of water, whereas the second one comes from the goethite to hematite formation plus OA degradation, and the last one extends till 800 °C and is assigned to thermal decomposition of organic chains into carbon.

**Figure 6:**
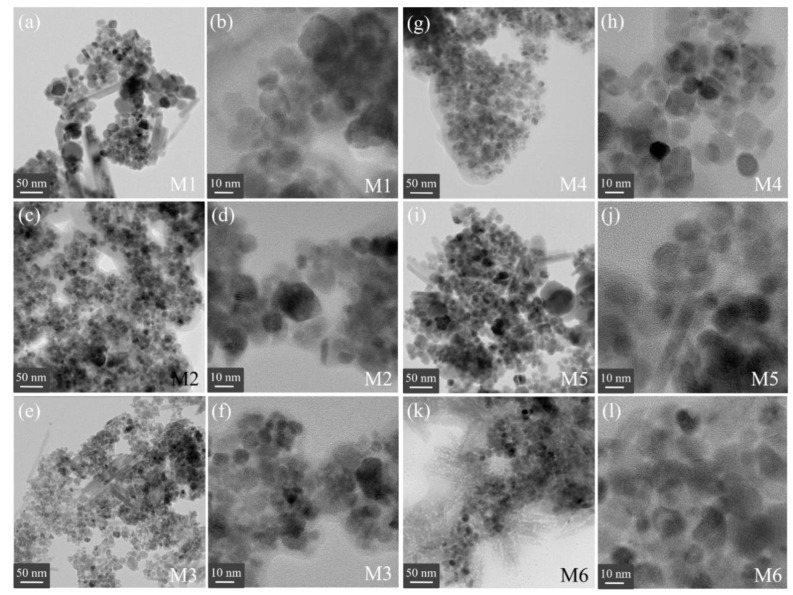
TEM images obtained for the M1–M6 samples. (**a**,**b**) are TEM images for M1 sample, (**c**,**d**) are TEM pictures for M2 sample, (**e**,**f**) are TEM images for M3 sample, (**g**,**h**) are TEM images for M4 sample, (**i**,**j**) are TEM pictures for M5 sample, while (**k**,**l**) are TEM magnifications for M6 sample.

In the case of 0.05 and 0.11 M for the M3 and M6 samples (CTAB), two marked mass losses can be described: (i) the first mass loss was assigned to dehydration of water attached to NPs surface and (ii) the second step, in the interval of 200 °C and 400 °C, happens faster and can be attributed to the endothermic CTAB organic decomposition (labeled with an arrow in Figure 6) [48]. This last assumption confirmed that the CTAB modifies the γ-Fe_2_O_3_ NPs surface produced at RT. We have assumed that the endothermic peak for the goethite phase is likely overlapped during CTAB decomposition. Hence, the total mass loss for both concentrations was found to be 16%.

### 2.5. TEM Analysis

Figure 6a–l depicts the TEM images for the M1–M6 samples. For each nanohybrid, it was possible to estimate the particle size distribution (PSD) as given in Figure S3. The PSD showed a control in the mean particle diameter, *<D>*, obtained in the range from 5 to 12 nm for all samples; see values in Table 1. As expected, the functionalization agent allowed controlling the PSD for samples prepared at RT, i.e., functionalized samples with particle sizes much smaller than 50 nm commonly found when a simple synthesis route is used at RT or even high temperatures [2,3]. In general, the M series depict spherical morphologies, which are assigned to nano γ-Fe_2_O_3_. However, it was noticed that needle-like or platelet morphologies were also obtained in some samples. According to the literature, they correspond to goethite-like morphologies [45]. The 11.1 nm M4 sample, corresponding to 2 µM PSS, has not this second goethite-like morphology; only spherical particles were noted. It means that a polymer environment influences the formation of pure γ-Fe_2_O_3_ seeds at RT, in contrast to the results after coating with organic oxalic acid and surfactant CTAB. Regarding the needle-like morphology of goethite, it can be attributed that their seeds are expected to form due to the highly alkaline medium, as reported by Ristic et al. [49], who used 3M of sodium hydroxide exposed for 24 h and days to form goethite needles.

### 2.6. EELS Analysis

**Figure 7:**
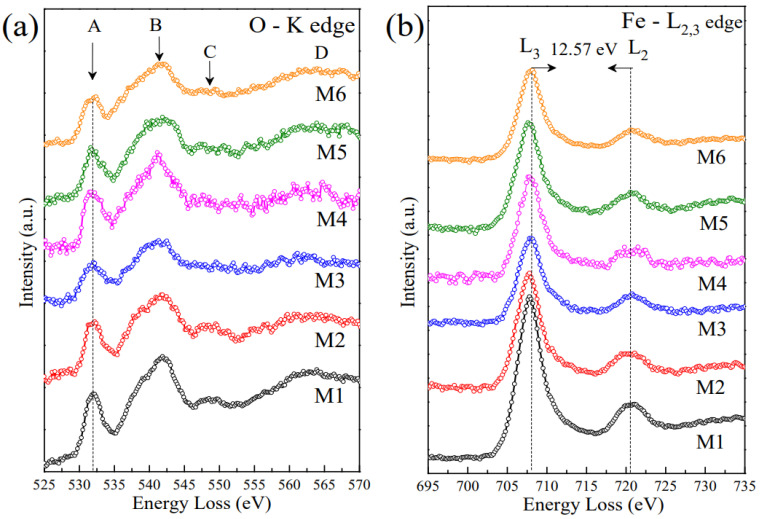
EELS spectra of the M1–M6 samples. (**a**) O-K edge and (**b**) Fe-Le_2,3_ edge.

Fe local environment and valence states can be first inferred and discerned from EELS analysis. Figure 7a,b shows the O-K edge and Fe-L_2,3_ edge. The distance between L_3_ and L_2_ is characteristic of iron-oxides, specifically from trivalent iron states [50,51]. In our case, a mean value of 12.6 eV was observed. Fe_3_O_4_ and γ-Fe_2_O_3_ have two featured peaks in the Fe-L_2,3_ edge [51], as compared to other iron-oxides, that often showed a shoulder below 710 eV. For instance, Chen et al. [50] studied the bulk and surface of iron-oxide NPs, and they differed in the appearance of other small peaks related to trivalent state and formation of goethite. In our case, no signal from the goethite phase was found on the bulk or surface of the NPs. This implies that the goethite is formed as a separated phase that is not directly interacting with the NPs surface. Therefore, no exchange bias effect is expected to be seen from *M(H)* curves due to the magnetic interaction between the ferrimagnetic γ-Fe_2_O_3_ NPs and antiferromagnetic α-FeOOH, as we will discuss below. By fitting the subtracted background spectra with a Gaussian component, the L_3_/L_2_ ratio was found to be (5.8 ± 0.4) eV [51]. This found value is similar to that obtained for γ-Fe_2_O_3_ NPs.

On the other hand, the O-K edge band has four defined positions [51]: (A) a peak at 530 eV that increases with the content of Fe_2_O_3_ phase, (B) the strongest peak located at 540 eV, (C) a weaker signal at 545–550 eV, and (D) a broad signal in the range 560–565 eV. In our case, the M series has all the four peaks. Nevertheless, both Fe_3_O_4_ and γ-Fe_2_O_3_ phases exhibit all above-mentioned peaks, making it difficult to differentiate them. However, the D peak is less intense and broader in the case of the γ-Fe_2_O_3_ phase than for the Fe_3_O_4_ phase. Importantly, the energy difference between B and A falls in the interval of (9.0–10.9) eV within an uncertainty of 0.4 eV. Therefore, this result suggests the presence of a residual goethite phase (α-FeOOH, no interacting with the NPs surface) or a core-shell-like Fe_3_O_4_@ γ-Fe_2_O_3_ arrangement [51].

### 2.7. Mössbauer Spectroscopy Analysis

**Figure 8:**
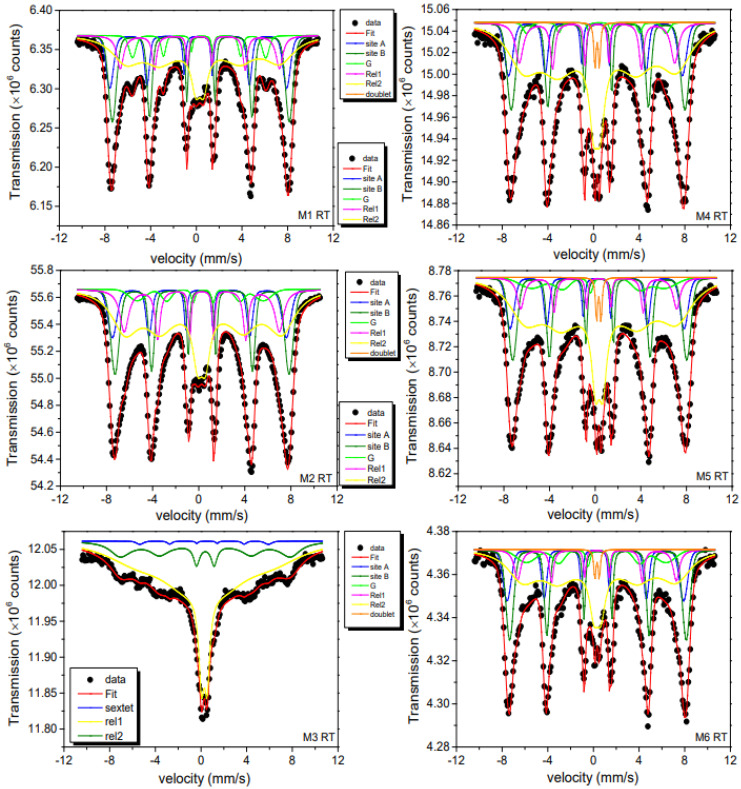
RT ^57^Fe Mössbauer spectra for the M series. The subspectra discussed in the text are also shown in the figure. Bottom boxes are the components belonging to the corresponding left fit spectrum while top boxes belong to the right fit spectrum. G stands for α-FeOOH phase.

**Figure 9:**
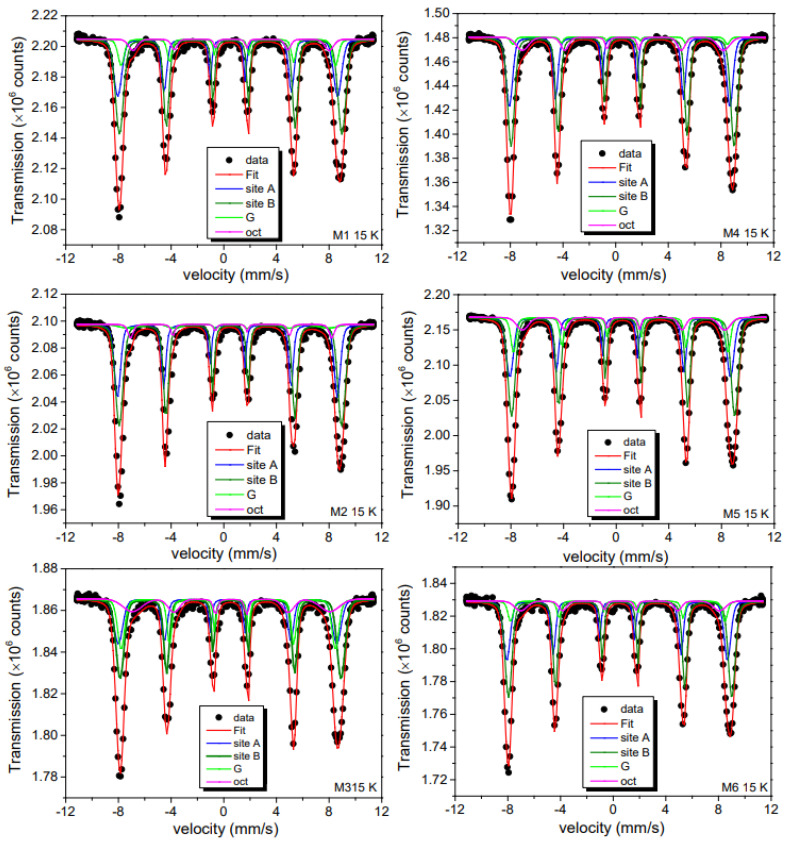
The 15 K ^57^Fe Mössbauer results for the M series. The subspectra discussed in the text are also shown in the figure. G stands for α-FeOOH phase.

RT and 15 K ^57^Fe Mössbauer spectra are respectively plotted in Figure 8 and Figure 9. Their broadened shapes indicate a superposition of hyperfine patterns due to several iron oxide phases. From a first visual inspection of the 15 K spectra with their asymmetric shape (lower velocity lines appear stronger compared to higher velocity), it is clear that the dominant spectral contribution comes from γ-Fe_2_O_3_. Spectra of pure Fe_3_O_4_ would reveal an asymmetry in the opposite direction due to the presence of divalent iron in parts of the B-sites with distinctly higher isomer shift [52]. RT spectra, in comparison, are still more complex due to the onset of spin dynamic fluctuations, leading to relaxation patterns that cannot be treated by the simple broadening of resonance absorption lines. This becomes most evident for the spectrum of the M3 sample where a partial collapse of the magnetic splitting is observed. Apart from these complications, the RT spectra reveal additional details that are not resolved clearly at 15 K. Most notable is a sub-pattern that can be associated with α-FeOOH, also detected by XRD. For disentangling this complex superposition of subspectra, we had to introduce a number of assumptions for reproducing the experimental data, yet admittedly limiting the meaning of a spectral “fit”. 

At RT, the spectrum is rather complex due to: (i) the contributions of the two iron-oxides phases (γ-Fe_2_O_3_ and α-FeOOH, labeled as G in Figure 8 and Figure 9) and (ii) the anisotropy overbarrier fluctuations of these phases, as it can be noticed by looking at the broadening effect of the spectra absorption lines and low field contributions at the central part of the RT Mössbauer spectra. 

The refined hyperfine parameters for the RT spectra are summarized in Table S5. For this analysis we have introduced six subspectra:

(i) Two sextet patterns with static hyperfine fields B_hf_ and Gaussian-shaped inhomogeneous hyperfine fine field distributions σ. These patterns are associated with Fe^3+^ in the A (tetrahedral) and B (octahedral) sites of γ-Fe_2_O_3_ in the magnetically blocked state. The ratio of spectral areas of A and B spectra could be kept fixed to the ideal one of 3/5, as found in the bulk crystalline compound. Magnetic hyperfine fields B_hf_, isomer shifts δ, and quadrupole splittings Q are close to those reported in literature [2,25,53,54].

(ii) A third component represents the secondary α-FeOOH phase identified by its hyperfine parameters, again in agreement with literature values. This component can be clearly seen in Figure 8 as a green-marked subspectrum. The relative absorption areas (RAA) of total spectral area ranges from 2–8%, values that agree with the results obtained from XRD measurements. This presence of the α-FeOOH phase was also supported by vibration IR and Raman analysis.
(iii) For reproducing the dynamic spectral parts caused by fluctuating magnetic hyperfine fields, we used, in a simplifying phenomenological approach, two relaxation patterns (Rel 1 and Rel 2) of Blume-Tjon type [55]. The dynamic aspects not being here in the center of the present discussion, we only mention that these spectra are typical for magnetically interacting NPs with fluctuation rates (see differing rate parameters γ_1_ and γ_2_ for up and down fluctuations between two levels) of the order of 8–9. Rel 2 (yellow) represents the small particles of the samples with its fraction varying from 42% to 79% of RAA. The highest fraction value is obtained for the 8.8 nm M3 sample, which showed the smallest particle size of the M series according to TEM data.

Rel 1 (magenta color) was associated with a smaller amount of uncompensated Fe^3+^ located at the NPs surface [12,53,54]. These are additional octahedral sites available for coordination with external molecules, i.e., favoring the functionalization process as discussed in reference [54]. In the center of the spectra of some samples there is a visible doublet pattern with an RAA of only a few percent that can be attributed to a small quantity of very small NPs with freely fluctuating superparamagnetic moments.

(iv) Finally, considering Raman results, we tried to include a possible spectral contribution by Fe_3_O_4_. Again, the hyperfine spectra of Fe_3_O_4_ can be clearly distinguished from those of γ-Fe_2_O_3_ at RT. All B-sites are now in a mixed valence state, resulting in an increased isomer shift value when compared to the pure γ-Fe_2_O_3_ phase. We, therefore, allowed for an additional subspectrum with the known fixed hyperfine parameters for nanomagnetite [52]. For the M1 and M2 samples, this component gave RAA values of (5 ± 2)%, while for the other samples, it was even lower, i.e., within fit uncertainty. Therefore, in general, due to above features one can assume that the magnetite layer is not homogenously covering the nanomaghemite surface.

At 15 K, spin relaxation is slowed down and the Mössbauer spectra could be fitted using 4 static but inhomogeneously broadened sextets: three components are related to γ-Fe_2_O_3_ (A and B sites) and α-FeOOH, as already discussed, and an additional sextet (“octahedral Fe^3+^”). Their hyperfine parameters are summarized in Table 2. The spectra of A and B sites are in excellent agreement with literature values for the inverse spinel crystalline γ-Fe_2_O_3_. The α-FeOOH patterns are not clearly resolved, but with the hyperfine parameters kept in agreement with the literature, we received relative areas very close to those derived from the XRD of Table 2. The relaxing components observed at RT have turned static and are adding now in part to the γ-Fe_2_O_3_ spectra. The additional sextet is contributed octahedral uncompensated Fe^3+^ spins, as already mentioned above.

We also considered the presence of a minor amount of nanomagnetite, as we observed at RT. However, at 15 K, this phase has a very complicated spectrum with six subspectra [52], three of them due to divalent iron with a distinct isomer shift of 0.9–1.0 mm/s. These could, however, not be resolved in our zero-field 15 K ^57^Fe Mössbauer spectra, though we cannot exclude small amounts within the uncertainties of RAA.

In brief, as we will see in the next section, there are indeed indications for the formation of a core-shell-like configuration involving possibly some Fe_3_O_4_ partially covering the γ-Fe_2_O_3_ core and yielding the observed final dark brown color. 

### 2.8. VSM Analysis

**Figure 10:**
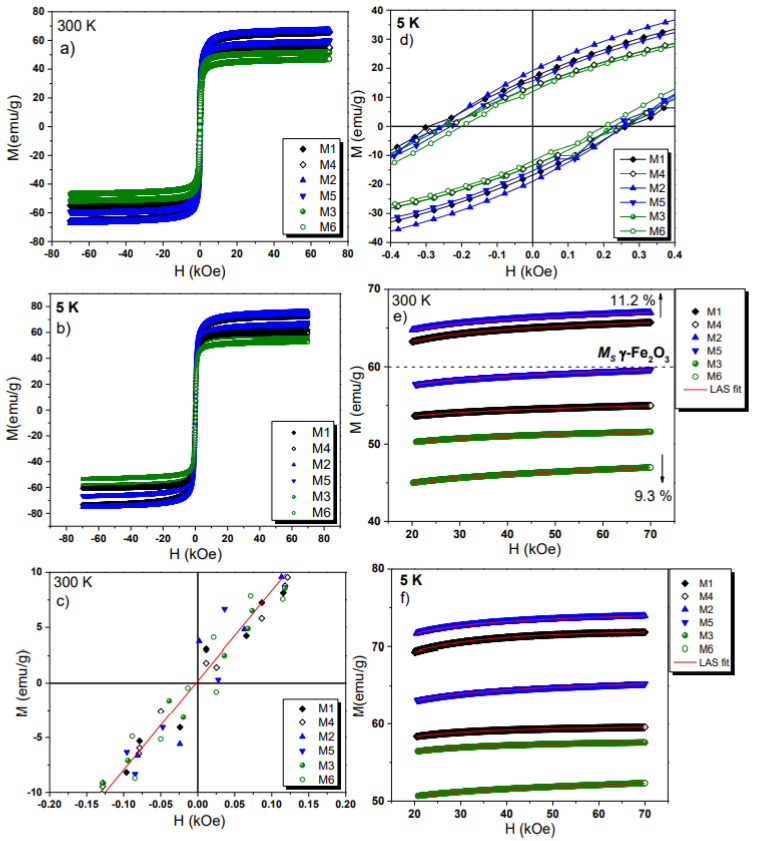
*M(H)* loops recorded at 300 K (**a**) and 5 K (**b**). Zoomed field section at 300 K (**c**) and 5 K (**d**). LAS fit at 300 K (**e**) and 5 K (**f**) for the M series samples.

*M(H)* loops were studied at 300 and 5 K, and the results are displayed in Figure 10a,b and zoomed regions in Figure 10c,d. As quantitative model for the high external field curves (20 to 70 kOe, see Figure 10e,f) we applied the Law of Approach to Saturation (LAS) equation [25]:

(1) $$M\left(H\right)=M_{s}\left(1-\frac{b}{H^{2}}\right)+\chi H\;$$

where *χ* is related to paramagnetic susceptibility contribution, while the *b* parameter relates the effective anisotropy constant (*K_eff_*) and saturation magnetization (*M_s_*) through the relation:

(2) $$b=\frac{4}{15}\frac{K_{eff}^{2}}{M_{s}^{2}}$$

The obtained fitting parameters are given in Table 3.

At 300 K, the *M_s_* of the M1 and M2 samples increases to ~67 emu g^−1^. This represents an enhancement of approximately 11% against that expected for a pure nano γ-Fe_2_O_3_ (60 emu g^−1^) [25]. These samples contain 1 µM of PSS and 14 mM of OA, respectively. The increase in *M_s_* value in this case when a small organic layer thickness is present (low organic concentrations), we interpreted as a possible formation of magnetite (Fe_3_O_4_) on the γ-Fe_2_O_3_ particle surfaces. The functionalization itself can hardly be responsible for the enhancement of *M_s_* because the organic materials are not ferromagnetic. They can help, however, to protect this core-shell arrangement from fast chemical oxidation. It is known that in the co-precipitation method, surface oxidation from Fe_3_O_4_ to γ-Fe_2_O_3_ occurs during the first days of synthesis [25]. Therefore, working with functionalization agents at RT during synthesis is a way of avoiding total oxidation; this last parameter will depend on organic layer nature and loading amount over the NPs as well. It is unlikely that the increase in *M_s_* value is related to an interaction of the α-FeOOH phase with the surface. From our observations, we can conclude that the α-FeOOH phase is separated from the NPs surface. Indeed, pure nanomagnetite is expected to have a *M_s_* value of ~90 emu g^−1^ [56]. Moreover, at high organic loading, the *M_s_* values will be reduced below reference values, and it will not be possible to say accurately that we have pure Fe_3_O_4_ or γ-Fe_2_O_3_. Therefore, we can assume a core-shell-like structure, where the core of the particle is the γ-Fe_2_O_3_ phase, and its shell is due to the Fe_3_O_4_ in total agreement with Raman, Mössbauer, and magnetization analysis. 

All the other samples M3–M6 exhibited smaller *M_s_* values, reaching a decrement of approximately 9% for the M6 sample. This reduction in *M_s_* values contrasts with the increase in organic layer thickness, as also reported in previous findings [2,11,53]. Hence, the core-shell configuration (magnetite contribution) cannot be totally confirmed for the samples M3–M6, in agreement with Mössbauer analysis.

All M samples keep a small residual coercivity of 50 Oe at 300 K, see Figure 10c, almost being in a superparamagnetic regime with small dipolar and exchange interactions. At 5 K (see Figure 10d), the M series shows ferrimagnetic character with increasing coercivity. This is in good agreement with the observed PSD obtained by TEM, showing a controlled size between 5 and 20 nm. The 5 K FC *M(H)* curves show an ordinary behavior with no horizontal loop shift effect that would be associated with the exchange bias effect. Exchange bias anisotropy could occur due to magnetic interactions, either between core and shell spins or, if present, a γ-Fe_2_O_3_-α-FeOOH interface. However, according to TEM data the α-FeOOH phase seems not have an interface with γ-Fe_2_O_3_ and the interparticle interactions are not resulting in a measurable exchange bias field. 

## 3. Methods and Materials

### 3.1. Synthesis and Functionalization of Magnetic Nanoparticles (NPs)

Six samples of γ-Fe_2_O_3_ NPs functionalized with sodium polystyrene sulfonate (nano γ-Fe_2_O_3_@PSS), oxalic acid (nano γ-Fe_2_O_3_@OA), and cetyltrimethylammonium bromide (nano γ-Fe_2_O_3_@CTAB) were synthesized each by the co-precipitation of 10.4 g of FeSO_4_·7H_2_O and 12 g of FeCl_3_ in alkaline medium with different functionalizing agent concentrations. The initial base solutions have been prepared by dissolving 250 mg of PSS (M1), 250 mg of OA (M2), 5 g of CTAB (M3), 500 mg of PSS (M4), 500 mg of OA (M5), and 10 g of CTAB (M6), in six different flasks with 100 mL of distilled water in each one. After 30 min of stirring at 350 rpm and at RT, the precursors were added to each solution and stirred for 10 min. Then, 25 mL of NH_4_OH were slowly poured over each mix (except M3, in which only 15 mL were poured) while stirring, and the black precipitates started to show up (black solution). Once each mixture accomplished homogeneity, the NPs were magnetically decanted and washed with distilled water until the pH became neutral. Finally, the samples were dried at 80 °C, pulverized in a mortar, and stored for characterizations. A final dark brown color was obtained for the M series. The calculated functionalizing agent molarity of each sample is 1 and 2 µM for the M1 and M4 (PSS) samples, 14 and 28 mM for the M2 and M5 (OA) samples, and 0.05 and 0.11 M for the M3 and M6 (CTAB) samples, respectively.

### 3.2. Characterization of Functionalized Nanohybrids

X-ray diffraction (XRD) data were taken in an Empyrean diffractometer using CuKα radiation at wavelength λ = 1.54056 Å (45 V, 40 mA). The XRD diffractograms were collected in the angle range of *2θ* = 10–80° with a fixed Bragg–Brentano geometry (steps of 0.01° and 10 s of counts per step). For crystallographic identification, the software Match v3 was employed. For Rietveld refinement the software FullProf Suite was used. Average particle size, particle distribution, and morphology were analyzed by electron imaging microscopy (EM) with two modes: transmission (TEM) and high-resolution (HRTEM) employing a 200 kV JEOL 2100F (Tokyo, Japan) instrument. The elemental compositions were investigated by electron energy-loss spectroscopy (EELS). EELS measurements were conducted in the scanning TEM imaging mode with a spot size of 0.7 nm, spectrometer aperture of 5 mm, and energy resolution of 1.8 eV. The infrared (IR) spectra were collected by an IRPrestige-21 Shimadzu spectrophotometer. The analyzed IR frequency range was in the interval of 400 to 4000 cm^−1^, with an optical resolution of 2 cm^−1^ at RT. The µ-Raman spectra were carried out at ambient conditions in a Renishaw inVia Raman microscope (Edinburgh, UK) in reflection geometry under 785 nm excitation wavelength with an initial laser power of 82.8 mW over the sample. The employed optical objective was of ×50 magnification. The protocol to increase the laser power had two steps: (i) measurements before burning that were performed with several fractions of initial laser power during 20 s of exposure, and (ii) after-burning µ-Raman measurements were performed, such as: the laser power percentage of 10% was first kept for 60 s of exposure, then the µ-Raman spectra were collected following step (i). Thermogravimetry (TG) measurements were taken in a Shimadzu equipment (Kyoto, Japan), the Mi (i = 1–6) samples were heated from RT to 800 °C in the presence of a synthetic air atmosphere (flux rate = 50 mL min^−1^) and heating rate of 10 °C/min. 

15 and 300 K ^57^Fe Mössbauer spectra were measured in transmission mode using a conventional spectrometer. A sinusoidal velocity sweep was used with a 40 mCi source of ^57^Co immersed in Rh matrix. For the low-temperature measurement, the source was kept at RT and the absorber was cooled down to 15 K using a Janis closed-cycle setup. Nylon sample holders were employed for the powder adsorbers with effective thicknesses equivalent to ca. 0.1 mg ^57^Fe per cm^2^. All isomer shifts (δ) are given relative to metallic iron at RT. Zero-field-cooling (ZFC) and warm field-cooling (WFC) magnetic hysteresis loops (*M(H)* loops) were recorded at RT and 5 K using a vibrating sample magnetometer (VSM) operated in a Dynacool setup for a maximum applied field of 70 kOe. The FC experiment was performed with a cooling field of 10 kOe and a sweep field of ±70 KOe (the 5 K FC experiments were performed to check for the existence or nonexistence of the exchange bias effect that may occur between core-shell spins or MNPs of ferrimagnetic (FI) γ-Fe_2_O_3_ and other magnetic structures of the nanohybrids).

## 4. Conclusions

We have been able to synthetize ferrimagnetic nanoparticles functionalized with different organic compounds [sodium polystyrene sulfonate (nano γ-Fe_2_O_3_@PSS), oxalic acid (nano γ-Fe_2_O_3_@OA), and cetyltrimethylammonium bromide (nano γ-Fe_2_O_3_@CTAB)] and with distinct layer thicknesses for these organic phases. The layer thickness is dependent on the organic concentration used during the co-precipitation route. More importantly, the co-precipitation process was performed at RT, and it favored a formation of functionalized γ-Fe_2_O_3_ NPs with sizes ca. 11 nm or smaller, which is four times smaller than similar nanoparticles prepared at RT without functionalization. From ^57^Fe Mössbauer data, it has been demonstrated that: (i) the functionalization occurs by octahedral Fe^3+^ spins on the particle surface and it reduces the spin relaxation effect even at 300 K, because no full collapse of sextets has been observed in Mössbauer spectra at this temperature, (ii) the core spins in the magnetically blocked state at 300 K can be mainly attributed to γ-Fe_2_O_3_, (iii) the presence of Fe_3_O_4_ shell for some samples (M1 and M2) and α-FeOOH phase in almost all other samples (except in sample M4). Samples prepared with PSS and OA have lower thickness of organic surface layer. For these samples, there was observed an increase in magnetization of approximately 11%, which could be attributed to the formation of Fe_3_O_4_ at the particle’s surfaces, which is supported by the results from Raman and Mössbauer measurements. Hence, due to their optimized magnetic properties, these functionalized NPs can find applications in the treatment of polluted effluents or other concerning environmental issues using combined magnetic separation and adsorption processes.

## Figures and Tables

**Table 1:** TEM values for mean particle diameter and D_m_ is the mode value obtained from the PSD histogram.

Sample	*<D>* (nm)	D_m_ (nm)	Standard Deviation
M1	12.2	11.5	2.95
M2	10.0	9.4	2.60
M3	8.8	8.4	1.88
M4	11.1	10.2	3.18
M5	13.8	13.1	3.29
M6	11.7	10.9	3.20

**Table 2:** Hyperfine parameters for the M samples at 15 K. RAA: relative spectral absorption area; δ: isomer shift vs. Fe at 300 K; B_hf_: Magnetic hyperfine field; Q: quadrupole splitting (fixed); σ: width of Gaussian distribution of B_hf_; W: Lorentzian width (fixed).

		RAA (%)	δ vs. Fe 300 K (mm/s)	B_hf_ (T)	Q (mm/s)	σ (T)	W (mm/s)
M1	γ-Fe_2_O_3_ A	31	0.30	51.9	0	1.6	0.24
	γ-Fe_2_O_3_ B	51	0.53	52.5	0	1.6	0.24
	α-FeOOH	11	0.47	50.6	−0.25	1.1	0.24
	Octahedral Fe^3+^	7	0.56	46.3	0	1.7	0.24
M2	γ-Fe_2_O_3_ A	34	0.28	51.9	0	1.1	0.24
	γ-Fe_2_O_3_ B	56	0.51	52.5	0	1.5	0.24
	α-FeOOH	5	0.47	47.5	−0.25	4.3	0.24
	Octahedral Fe^3+^	6	0.56	47.5	0	0.9	0.24
M3	γ-Fe_2_O_3_ A	24	0.29	51.6	0	1.7	0.24
	γ-Fe_2_O_3_ B	40	0.52	52.2	0	1.5	0.24
	α-FeOOH	22	0.47	50.6	−0.25	1.3	0.24
	Octahedral Fe^3+^	14	0.56	46.6	0	4.2	0.24
M4	γ-Fe_2_O_3_ A	32	0.30	52.0	0	1.2	0.24
	γ-Fe_2_O_3_ B	53	0.53	52.6	0	1.3	0.24
	α-FeOOH	3	0.47	50.6	−0.25	0.8	0.24
	Octahedral Fe^3+^	11	0.56	47.5	0	2.6	0.24
M5	γ-Fe_2_O_3_ A	29	0.30	52.1	0	1.4	0.24
	γ-Fe_2_O_3_ B	48	0.53	52.7	0	1.4	0.24
	α-FeOOH	14	0.47	50.6	−0.25	0.9	0.24
	Octahedral Fe^3+^	9	0.56	47.7	0	2.5	0.24
M6	γ-Fe_2_O_3_A	31	0.30	52.1	0	1.3	0.24
	γ-Fe_2_O_3_B	52	0.53	52.7	0	1.3	0.24
	α-FeOOH	9	0.42	50.6	−0.25	0.9	0.24
	Octahedral Fe^3+^	9	0.54	46.9	0	2.6	0.24
error		±3	±0.02	±0.1		±0.1	

**Table 3:** Fit parameters from the LAS equation at high magnetic fields (70 kOe).

**300 K**			
Sample	*M_s_* (emu g^−1^)	*b* (kOe)^2^	*χ* (emu/gOe)
M1	65.8 (2)	0.84 (5)	0.01 (2)
M2	66.7 (2)	0.67 (5)	0.01 (2)
M3	51.2 (2)	0.46 (5)	0.01 (2)
M4	54.4 (2)	0.38 (5)	0.01 (2)
M5	58.9 (1)	0.56 (5)	0.02 (2)
M6	45.9 (2)	0.59 (5)	0.02 (2)
**5 K**			
M1	73.3 (2)	1.05 (3)	0.005 (1)
M2	74.6 (2)	0.82 (3)	0.003 (1)
M3	57.9 (2)	0.53 (3)	0.002 (3)
M4	59.9 (2)	0.55 (3)	0.001 (5)
M5	65.4 (2)	0.76 (3)	0.009 (3)
M6	51.6 (2)	0.49 (3)	0.016 (3)

## Data Availability

The original data related to this research can be requested at any time from the corresponding author’s email: juan.ramos5@unmsm.edu.pe.

## Supplementary Materials

The supporting information can be downloaded at: https://www.mdpi.com/article/10.3390/ijms23158279/s1. References [57,58] are cited in the Supplementary Materials.
