# Supplementary material for: Progress toward Room-Temperature Synthesis and Functionalization of Iron-Oxide Nanoparticles
Source: Int J Mol Sci. 2022 Jul 27;23(15):8279. doi: 10.3390/ijms23158279 (PMC9368286; doi:10.3390/ijms23158279)
Supplement: Supplementary file 1 [file ijms-23-08279-s001.zip › ijms-1834426-supplementary.pdf]

International Journal of *Molecular Sciences*

Supplementary materials

Progress toward Room-Temperature Synthesis and Functionalization of Iron-Oxide Nanoparticles

Diego A. Flores-Cano [1], Noemi-Raquel Checca-Huaman [2], Isabel-Liz Castro-Merino [2], Camila N. Pinotti [3], Edson C. Passamani [3], Fred Jochen Litterst [4] and Juan A. Ramos-Guivar [1,*]

1 Grupo de Investigación de Nanotecnología Aplicada para Biorremediación Ambiental, Energía, Biomedicina y Agricultura (NANOTECH), Facultad de Ciencias Físicas, Universidad Nacional Mayor de San Marcos, Av. Venezuela Cdra 34 S/N, Ciudad Universitaria, Lima 15081, Peru; diego.flores4@unmsm.edu.pe
2 Centro Brasileiro de Pesquisas Físicas (CBPF), R. Xavier Sigaud, 150, Urca, Rio de Janeiro 22290-180, Brazil; noemiraquelchecca@gmail.com (N.-R.C.-H.); isabel5cas@hotmail.com (I.-L.C.-M.)
3 Physics Department, Federal University of Espírito Santo, Vitória 29075-910, Brazil; camilapinotti23@gmail.com (C.N.P.); passamaniec@yahoo.com.br (E.C.P.)
4 Institut für Physik der Kondensierten Materie, Technische Universität Braunschweig, 38106 Braunschweig, Germany; j.litterst@tu-braunschweig.de
* Correspondence: juan.ramos5@unmsm.edu.pe; Tel.: +51-1-914728212

Supplementary Figures

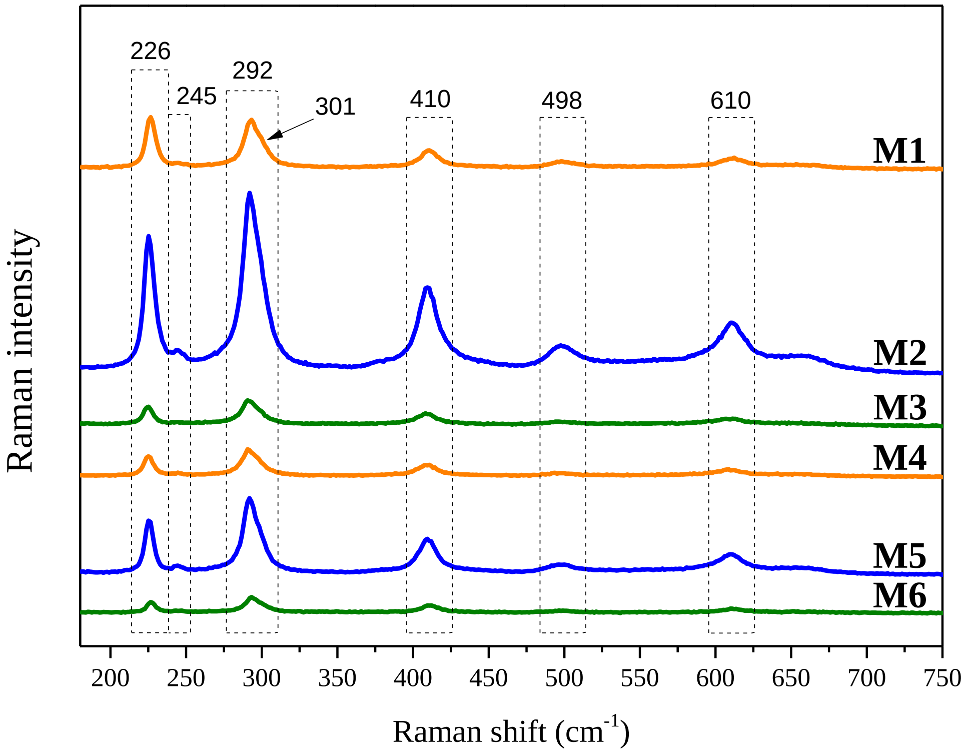

Figure S1. Raman spectra at 8.28 mW after the burning of the M1-M6 samples. The apparent band positions are indicated in cm^{-1}.

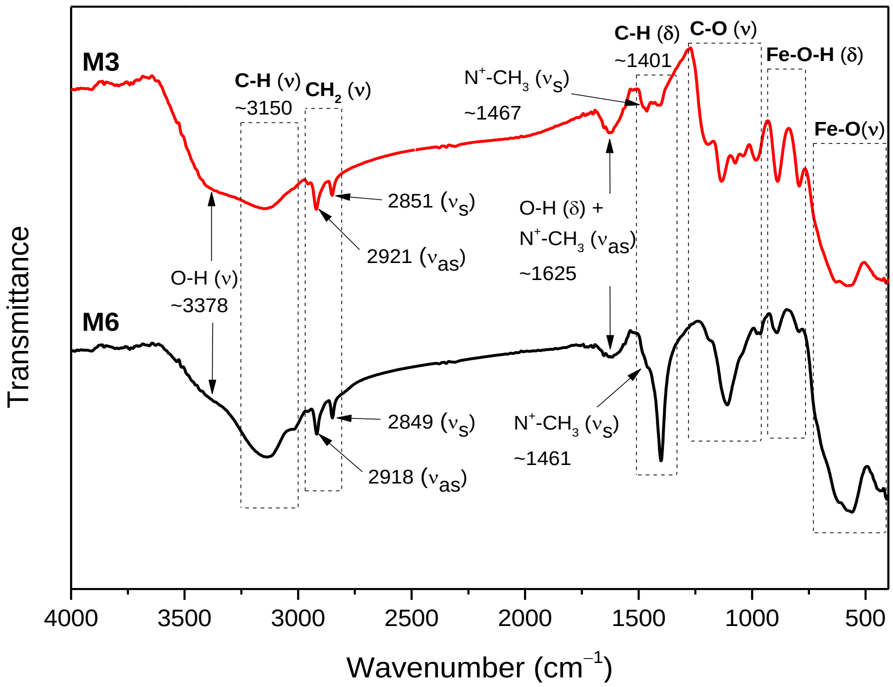

Figure S2. FTIR spectra of the M3 and M6 samples. The peaks positions are indicated in cm^{-1}.

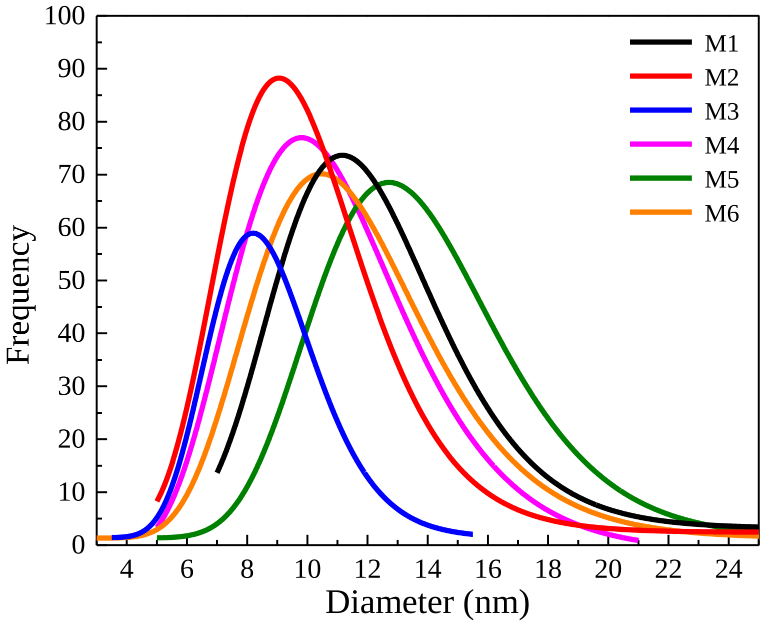

Figure S3. PSD for the M series samples.

Supplementary Tables

Table S1. Refinement and statistical parameters, R_p (%) as profile refinement, R_{wp} (%) (weighted profile residual), R_{exp} (%) as expected profile residual, and Goodness of fit, χ^2, of the refined samples M1-M6.

Samples	Phases	Cagliotti Parameters			Lattice Parameters (Å)	Statistical Parameters
		U	V	W		
M1	γ-Fe_2O_3	0.83	-0.57	0.36	a = b = c = 8.40<br>α = β = γ = 90°	R_p = 73.87<br>R_{wp} = 32.74<br>R_{exp} = 29.34<br>χ^2 = 1.24
	α-FeOOH	1.08	-1.08	0.21	a =4.63, b =10.03<br>c = 3.04<br>α = β = γ = 90°	
M2	γ-Fe_2O_3	0.80	-0.80	0.25	a = b = c =8.41<br>α = β = γ = 90°	R_p = 63.81<br>R_{wp} = 30.25<br>R_{exp} = 29.48<br>χ^2 = 1.05
	α-FeOOH	0.42	-0.07	0.95	a =4.59, b =10.00 c = 3.03<br>α = β = γ = 90°	
M3	γ-Fe_2O_3	0.44	-0.36	0.14	a = b = c = 8.41<br>α = β = γ = 90°	R_p = 66.39<br>R_{wp} =36.72<br>R_{exp} = 36.15<br>χ^2 = 1.03
	α-FeOOH	0.38	-0.47	0.33	a =4.65, b =10.03 c = 3.04<br>α = β = γ = 90°	
M4	γ-Fe_2O_3	0.13	-0.27	0.17	a = b = c = 8.41<br>α = β = γ = 90°	R_p = 99.14<br>R_{wp} =41.73<br>R_{exp} = 41.69<br>χ^2 = 1.00
M5	γ-Fe_2O_3	0.24	-0.37	0.17	a = b = c = 8.40<br>α = β = γ = 90°	R_p = 108.75<br>R_{wp} = 47.57<br>R_{exp} = 45.85<br>χ^2 = 1.08
	α-FeOOH	0.24	-1.71	1.04	a =4.63, b =10.05 c = 3.04<br>α = β = γ = 90°	
M6	γ-Fe_2O_3	0.63	-0.36	0.24	a = b = c = 8.40<br>α = β = γ = 90°	R_p = 93.58<br>R_{wp} = 42.42<br>R_{exp} = 38.85<br>χ^2 = 1.19
	α-FeOOH	1.42	-0.41	0.81	a =4.59, b =10.00 c = 3.03<br>α = β = γ = 90°	

Table S2. Microstructural parameters of the samples M1-M6. D_{XRD} is the mean crystallite diameter.

Samples	Phases	D_{XRD} (nm)	Microstrain (%)	Phase contribution (%)
M1	γ-Fe_2O_3	8.90	42.27	90
	α-FeOOH	14.31	48.23	10
M2	γ-Fe_2O_3	9.67	41.64	95
	α-FeOOH	4.81	78.95	5
M3	γ-Fe_2O_3	7.72	32.75	81
	α-FeOOH	13.91	28.61	19
M4	γ-Fe_2O_3	10.27	16.68	100
M5	γ-Fe_2O_3	12.21	27.66	90
	α-FeOOH	12.33	22.72	10
M6	γ-Fe_2O_3	10.33	48.08	91
	α-FeOOH	10.60	179.86	9

Table S3. Raman shift values of the vibrational modes of each phase of the M1-M6 samples at different laser power values.

Sample	Laser power (mW)	Raman shift (cm^{-1})			
		γ-Fe_2O_3	α-FeOOH	α-Fe_2O_3	PSS
M1	4.14	372, 498, 674	244, 299	-	1130
	8.28	365, 491, 690	247, 305	616	1045, 1130
M2	4.14	369, 492, 680	-	-	
	8.28	487, 671	-	217, 281, 391, 601	
M3	0.41	356, 502, 690	308, 384	-	
	0.83	370, 514, 692	250, 304, 389	-	
M4	0.41	366, 503, 681	-	-	1123, 1598
	0.83	350, 500, 690	-	-	1123, 1596
M5	0.83	371, 495, 693	243, 303	-	
	4.14	351, 492, 687	-	220, 288	
M6	0.41	355, 490, 683	255, 300, 385	-	
	0.83	355, 489, 680	250, 302, 385	-	

Table S4. Estimated positions of the observed vibration bands at each type of functionalized nano γ-Fe_2O_3 sample.

Wavenumber (cm^{-1})			Assigned bands	References
@PSS	@OA	@CTAB		
3390	3384	3378	O-H stretching vibrations	[33,57,58]
3124	3156	3150	C-H stretching vibrations	[37]
2916	-	2921, 2918	CH2 asymmetrical stretch	[31,38,39]
2851	-	2851, 2849	CH2 symmetrical stretch	[31,38,39]
-	1661	-	C=O	[33]
1619	1661	1625	O-H bending	[14,34]
		1625	N^+-CH3 asymmetrical vibration	[39]
	1568, 1565		COO asymmetrical stretch	[33,34]
		1467	N^+-CH3 symmetrical vibration	[39]
	1402, 1413		COO symmetrical stretch	[33,34]
1400	1402, 1413	1401	C-H bending vibration + N bonded ion groups	[40]
1007, 1037, 1124, 1180, 1128	-	-	–SO3 stretching vibrations	[30–32]
-	1046, 1126, 1278, 1281	981, 1040, 1075, 1109, 1135, 1193, 1200	C-O stretching vibrations	[14,37,39,42]
795, 890	788, 791, 889	793, 887	Fe-O-H bending vibration	[27–29]
437, 567, 630	438, 565, 630	432, 562, 629	Fe-O stretching vibrations	[25,26]

Table S5. Hyperfine parameters for the M series recorded at RT. RAA: relative spectral absorption area; δ: isomer shift vs Fe at 300K; B_{hf}: Magnetic hyperfine field; Q: quadrupole splitting; σ: width of Gaussian distribution of B_{hf}; log γ_1, log γ_2: ^{10}log of fluctuation rates (see main text); W: Lorentzian width (fixed). Spectra of M4, M5, and M6 reveal an additional doublet component of very fast fluctuating spins.

		RAA (%)	δ (mm/s)	Bhf (T)	Q (mm/s)	σ (T)	log γ1	log γ2	W (mm/s)
M1	γ-Fe_2O_3 A	15	0.17	48.2	0	1.7			0.24
	γ-Fe_2O_3 B	25	0.40	48.2	0	1.7			0.24

	α-FeOOH	6	0.37	36.0	-0.26	1.5			0.24
	Rel 1	11	0.30	45	0		9.1	7.6	0.24
	Rel 2	42	0.44	44	0.3		8.2	8.0	0.24
M2	γ-Fe_2O_3 A	12	0.17	46.9	0	2.1			0.24
	γ-Fe_2O_3 B	21	0.40	46.9	0	1.9			0.24
	α-FeOOH	5	0.37	34.0	-0.26	4.6			0.24
	Rel 1	14	0.38	45.0	0		9.2	7.9	0.24
	Rel 2	48	0.43	44.0	0.15		8.2	8.0	0.24
M3	Sextet	1	0.40	35	0	1.5			0.24
	Rel 1	20	0.38	48	0		8.4	7.9	0.24
	Rel 2	79	0.29	48	0		8.6	8.4	0.24
M4	γ-Fe_2O_3 A	12	0.17	47.2	0	1.9			0.24
	γ-Fe_2O_3 B	20	0.40	47.2	0	2.6			0.24
	α-FeOOH	2	0.37	38.0	-0.26	0.8			0.24
	Rel 1	11	0.31	45.0	0		9.2	7.8	0.24
	Rel 2	54	0.44	44.0	0.25		8.2	8.0	
	Doublet	2	0.32		0.39				0.21
M5	γ-Fe_2O_3 A	11	0.17	47.4	0	1.9			0.24
	γ-Fe_2O_3 B	19	0.40	47.4	0	2.0			0.24
	α-FeOOH	7	0.37	36.0	-0.26	6.3			0.24
	Rel 1	8	0.31	45.0	0		9.2	7.8	0.24
	Rel 2	54	0.44	44.0	0.15		8.3	8.1	0.24
	Doublet	2	0.32		0.39				
M6	γ-Fe_2O_3 A	14	0.17	48.1	0	1.9			0.24
	γ-Fe_2O_3 B	24	0.40	48.1	0	1.7			0.24
	α-FeOOH	8	0.37	38.0	-0.26	5			0.24
	Rel 1	9	0.31	45.0	0		9.1	7.6	0.24
	Rel 2	44	0.44	44.0	0.15		8.2	8.0	
	Doublet	1	0.32		0.39				0.21
**Errors sextets**		±3	±0.02	±0.1	±0.02	±0.1	±0.1	±0.1	
**Errors doublet**		±1	±0.02		±0.02				±0.02

Supplementary References

57. Neto, W.S.; Dutra, G.V.S.; Jensen, A.T.; Araújo, O.A.; Garg, V.; de Oliveira, A.C.; Valadares, L.F.; de Souza, F.G., Jr.; Machado, F. Superparamagnetic nanoparticles stabilized with free-radical polymerizable oleic acid-based coating. *J. Alloys Compd.* **2018**, *739*, 1025–1036.
58. Soares, P.P.; Barcellos, G.S.; Petzhold, C.L.; Lavayen, V. Iron oxide nanoparticles modified with oleic acid: Vibrational and phase determination. *J. Phys. Chem. Solids* **2016**, *99*, 111–118.
